# Sympatric and Allopatric Divergence of MHC Genes in Threespine Stickleback

**DOI:** 10.1371/journal.pone.0010948

**Published:** 2010-06-16

**Authors:** Blake Matthews, Luke J. Harmon, Leithen M'Gonigle, Kerry B. Marchinko, Helmut Schaschl

**Affiliations:** 1 Aquatic Ecology Department, Swiss Federal Institute of Aquatic Science and Technology (EAWAG), Kastanienbaum, Switzerland; 2 Department of Biological Sciences, University of Idaho, Moscow, Idaho, United States of America; 3 Zoology Department, University of British Columbia, Vancouver, Canada; 4 Fred Hutchinson Cancer Research Center, Seattle, Washington, United States of America; 5 Research Institute of Wildlife Ecology, University of Veterinary Medicine, Vienna, Austria; University of Texas Arlington, United States of America

## Abstract

Parasites can strongly affect the evolution of their hosts, but their effects on host diversification are less clear. In theory, contrasting parasite communities in different foraging habitats could generate divergent selection on hosts and promote ecological speciation. Immune systems are costly to maintain, adaptable, and an important component of individual fitness. As a result, immune system genes, such as those of the Major Histocompatability Complex (MHC), can change rapidly in response to parasite-mediated selection. In threespine stickleback (*Gasterosteus aculeatus*), as well as in other vertebrates, MHC genes have been linked with female mating preference, suggesting that divergent selection acting on MHC genes might influence speciation. Here, we examined genetic variation at MHC Class II loci of sticklebacks from two lakes with a limnetic and benthic species pair, and two lakes with a single species. In both lakes with species pairs, limnetics and benthics differed in their composition of MHC alleles, and limnetics had fewer MHC alleles per individual than benthics. Similar to the limnetics, the allopatric population with a pelagic phenotype had few MHC alleles per individual, suggesting a correlation between MHC genotype and foraging habitat. Using a simulation model we show that the diversity and composition of MHC alleles in a sympatric species pair depends on the amount of assortative mating and on the strength of parasite-mediated selection in adjacent foraging habitats. Our results indicate parallel divergence in the number of MHC alleles between sympatric stickleback species, possibly resulting from the contrasting parasite communities in littoral and pelagic habitats of lakes.

## Introduction

Competition and predation are central mechanisms of ecological speciation [1], [2], but the role of parasitism in the evolution of host reproductive incompatability is less clear [3]–[5]. Parasites readily shape, by natural selection, the phenotype and genotype distributions of their hosts [6], [7], and can promote host genetic diversity via balancing [8] and disruptive selection [9]. Theory suggests that speciation is more likely when functional traits [10], such as those underlying ecological performance, are under both divergent natural selection and sexual selection [11]. Such traits have been dubbed ‘magic traits’ [11], and are common elements of sympatric speciation models [5], [12]. Parasites are known to cause strong selection on several host traits associated with mate choice [4], [6], [13], such as body size [14] and odor [15]. However, from an empirical perspective, the role of parasites in host speciation is highly understudied [4], and so the consequences of parasite-mediated selection for host diversification remain uncertain [5], [12].

In theory, parasites can cause divergent selection on their hosts by affecting tradeoffs between host life history and immune defense [16]–[18]. The vertebrate immune system consists of two components, namely the innate immune system, which includes several non-specific mechanisms to protect hosts from infection [19], and the adaptive immune system, which targets specific pathogens and is driven by the extremely polymorphic genes of the Major Histocompatibility Complex (MHC) [20]. Because immune systems are costly to maintain [21], the optimal allocation of energy to immune defense is strongly dependent on environmental conditions and species interactions [17], [18], [22]. In aquatic environments, parasite risk varies both at the regional scale, for example between lake and river environments [22], and at the local scale, for example between adjacent foraging habitats in lakes [23]–[25]. As a result, spatial variation in the nature of antagonistic coevolutionary interactions between hosts and parasites [26] could promote divergence in hosts' strategies of energy allocation toward adaptive and innate immune defenses [16] and lead to divergence in hosts' MHC genotypes [12]. Divergence in immune system genes that underly proximate mating cues, such as odor [27], could ultimately influence reproductive isolation between sympatric species [28].

Parasitism can be a persistent selective force in freshwater fish populations [24] and may drive adaptive divergence between sympatric fish species [12], [14], [28]. Sticklebacks, for example, are infected by a diverse range of parasites, including species from the taxon *Mollusca*, *Crustacea*, *Nematoda*, *Cestoda*, *Trematoda* and others [25], and their parasite-load can reflect sex- and individual-based habitat specialization [23], [25], [29]. Parasite-mediated selection has been implicated in the divergence of MHC genes between river and lake stickleback populations in Northern Germany [30] and is thought to influence sexual selection [15], [31] and play a role in ecological speciation [12]. In fact, MHC genotype has been proposed as a magic-trait in stickleback [12], partly because immune system genes can play a dual role in both parasite resistance and mate choice [15]. In general, however, little is known about the strength and form of natural selection (e.g. divergent, directional, or balancing) that parasites exert on host populations.

We use threespine stickleback to investigate the potential importance of parasites and immune systems for ecological speciation [1], [12]. In several British Columbia lakes, threespine stickleback have undergone a recent parallel diversification, resulting in a limnetic species that specializes on plankton in open water habitats and a benthic species that specializes on macro-invertebrates in littoral habitats [32], [33]. Most speciation research on stickleback has focussed on the role of competition and predation in generating divergent selection regimes within lakes [1]. More recently, researchers have found that sympatric stickleback species have different parasite communities [25], but the importance of parasitism for ecological speciation in stickleback remains unclear [25]. Limnetics are more frequently parasitized by species using planktonic crustacea as intermediate hosts (e.g. *Schistocephalus solidus*), whereas, benthics are more commonly parasitized by species using snails as intermediate hosts [25]. A recent study speculated that contrasting parasite-mediated selection regimes in pelagic and littoral habitats of lakes could cause divergence in the MHC alleles between limnetic and benthic sticklebacks [12]. Here, we examine whether the proximate foraging environment used by sticklebacks is predictive of their MHC genotype, and test the hypothesis that benthic and limnetic sticklebacks have divergent MHC genotypes. We use a simulation model to examine how the strength of assortative mating, along with parasite-mediated selection in pelagic and littoral habitats, could affect the distribution of MHC alleles in sympatric stickleback species.

## Results

### MHC allele number and composition

Overall, we found 56 unique alleles in our MHC analysis of 342 stickleback (Kennedy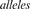 = 14; Cranby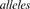 = 30; Priest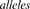 = 50; Paxton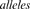 = 37). At the population level, we found that the limnetics tended to have lower allelic richness than benthics in both Paxton Lake and Priest Lake (Table 1, AR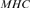). The Cranby population had an intermediate allelic richness in relation to benthics and limnetics in Paxton Lake, but a lower richness in relation to both species in Priest Lake (Table 1). The Kennedy Lake population had the lowest allelic richness overall.

**Table 1:** Allelic richness of MHC and microsatellites.

Population	N 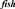	MHC 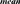	MHC 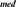	MHC 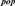	AR 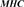	H 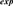	H 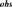	Rs	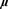 Sats 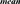
Kennedy pelagic	54	2.2	2	14	0.4	–	–	–	–
Cranby intermediate	96	4.3	4	27	1.1	0.62	0.46	9.6	1.54
Paxton benthic	48	4.4	5	36	1.2	0.60	0.48	8.0	1.50
Paxton limnetic	46	2.9	4	24	0.9	0.67	0.55	9.8	1.54
Priest benthic	48	5.0	4.5	37	1.5	0.76	0.66	11.6	1.67
Priest limnetic	48	3.9	3	24	1.4	0.69	0.56	11.9	1.60

Summary of the mean (MHC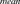) and median (MHC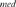) number of MHC class IIB alleles per individual, and the number of different alleles found in the sampled population (MHC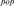). The number of alleles detected per sampled stickleback (AR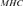) is a standardized index of population-level allelic richness, and was calculated via bootstrapping with a constant sampling effort (N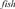 = 20). Limnetics and benthics did not differ in the levels of heterozygosity (H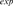 and H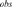), or in the mean number of alleles per microsatellite locus, calculated at both the population level (Rs) and at the individual level (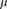
Sats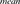).

**Figure 1:**
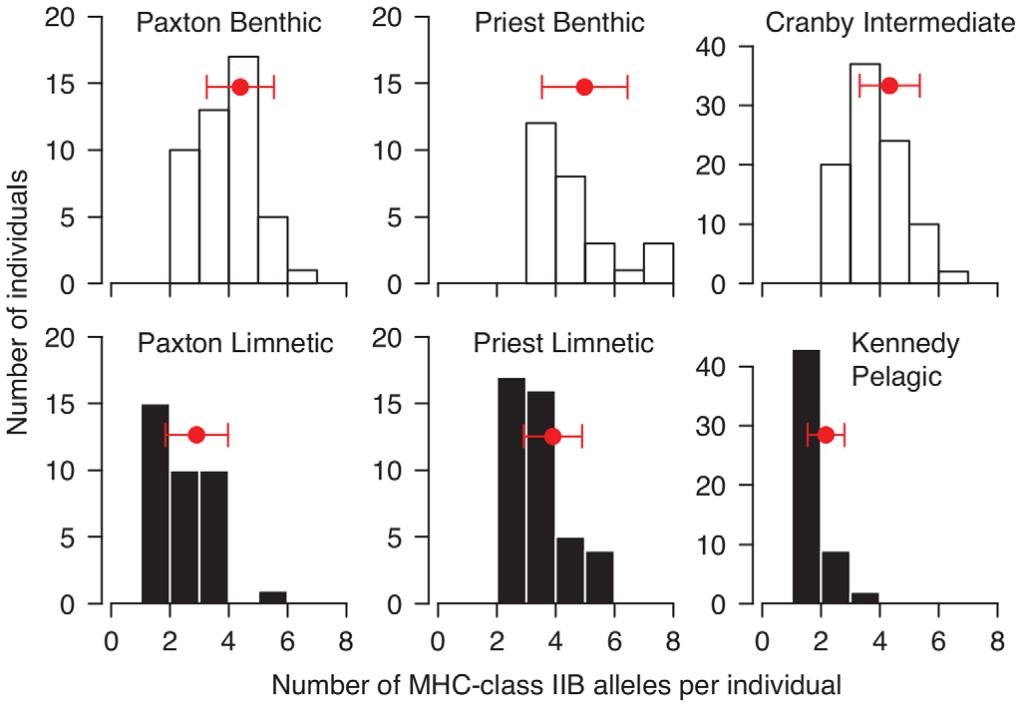
Frequency distributions of the number of MHC-class IIB alleles per individual. The top panels show the distribution of littoral and intermediate eco-types, and the bottom panels show the distribution of pelagic eco-types. Red points and bars indicate the mean population allelic richness (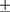 SD).

At the individual level, we found that limnetics had lower allelic richness than benthics in both Paxton (t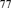 = −6.1, p
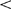
0.001) and Priest Lake (t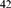 = −3.4, p
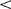
0.001, Table 1, Figure 1). Cranby sticklebacks had an intermediate allelic richness in relation to limnetics and benthics in Priest (Limnetics: t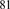 = −2.3, p
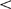
0.02, Benthics: t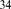 = 2.2, p
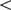
0.04), but had a similar allelic richness as Paxton benthics (Limnetics: t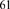 = −6.9, p
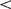
0.001, Benthics: t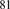 = 0.3, p
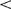
0.77). Kennedy Lake sticklebacks, consistent with their pelagic phenotype and diet, had the lowest allelic richness per individual (all pair-wise t-tests: p
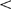
0.001). Overall, these results suggest there has been parallel divergence in the number of MHC alleles per individual between stickleback populations foraging in littoral and pelagic habitats (Figure 1).

**Figure 2:**
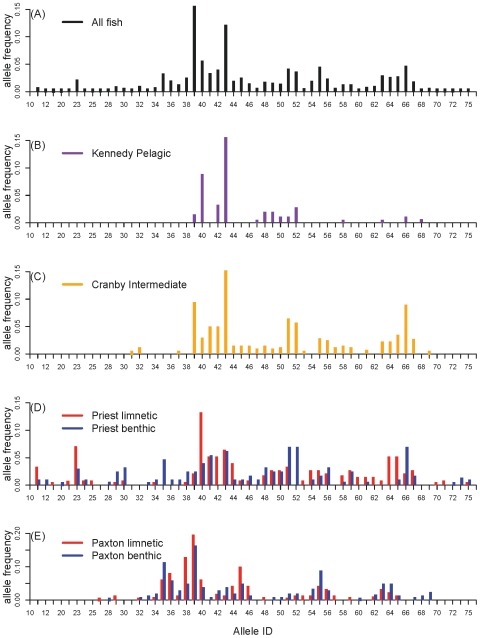
Frequency distributions of MHC-class IIB alleles in each population. Panel (A) is the distribution for all the alleles identified in the study. Panel (B) is the distribution for the pelagic phenotype in Kennedy Lake. Panel (C) is the distribution for the intermediate phenotype in Cranby Lake. Panels (D) and (E) are the distributions for each stickleback species in the two lakes with species pairs.

The log-linear analysis strongly supported six of our seven hypotheses about how allele number and frequency differed among populations, and between ecotypes within lakes (Tables 2 and 3). We classified the model results into five groups, A (high AIC) through E (low AIC), which were ordered by their increasing support from the data. The poor support for A-models, relative to all other models, suggests there has been significant divergence in MHC allele frequencies that cannot be accounted for by differences in allele number among populations (Table 2). The greater support for the C-models over the B-Models, indicates that within sympatric lakes limnetics and benthics differ in their allele frequencies. However, the greater support for D- and E-models indicates that stickleback populations in different lakes have contrasting allele frequencies, suggesting that the divergence in allelic composition (i.e. the identities of the alleles) has not occurred in parallel in both lakes with species pairs. The best model (E-3), considers all the two-way interactions between ‘Allele’, ‘Lake’, and ‘Ecotype’ (Table 2). Overall, these results suggest that the number of MHC alleles has diverged in parallel between benthics and limnetics (Figure 1), but the particular alleles involved in the divergence has differed between lakes with species pairs (Table 2, Figure 2).

**Table 2:** Summary of log-linear models.

Model ID	Allele	Lake	Ecotype	Allele* lake	Allele*ecotype	Lake*ecotype	Allele*lake*ecotype	n params	AIC	dAIC	Weight
A-1		x						3	7488	1784	0
A-2		x	x					4	7481	1777	0
A-3			x					3	7479	1775	0
A-4		x	x			x		5	7456	1752	0
B-1	x							56	6293	589	0
B-2	x	x						58	6273	569	0
B-3	x		x					58	6263	559	0
B-4	x	x	x			x		60	6237	533	0
C-1	x	x	x		x			169	5986	282	0
C-2	x		x		x			168	5985	281	0
C-3	x	x	x		x	x		170	5959	255	0
D-1	x	x		x				168	5757	53	0
D-2	x	x	x	x				169	5749	45	0
E-1	x	x	x	x	x			224	5726	22	0
E-2	x	x	x	x		x		170	5719	15	0
E-3	x	x	x	x	x	x		225	5704	0	1
E-4	x	x	x	x	x	x	x	280	5748	44	0

We compared the fit of seventeen possible models for our data, considering all combinations of single factor effects with two- and three-way interactions. For each model, an x denotes that a given factor or interaction was included. The number of parameters for each model is given. In some cases, certain parameters were redundant, and are not included in this count. AIC scores, delta-AIC values, and Akaike weights are given in the last three columns. A model including all two-way interactions but no three-way interaction term is strongly favored by the data.

**Table 3:** Conclusions from the log-linear analysis in Table 2.

Model Effect	Hypothesis being tested by the model formulation	Supported?
Lake	Does the average number of alleles differ among lakes?	Yes
Ecotype	Does the average number of alleles differ among ecotypes?	Yes
Allele	Do alleles have different frequencies?	Yes
Allele * lake	Do lakes have different allele frequencies?	Yes
Allele * ecotype	Do ecotypes have different allele frequencies?	Yes
Lake * ecotype	Does the average number of alleles between ecotypes differ among lakes?	Yes
Allele * lake * ecotype	Does each ecotype in each lake have different allele frequencies?	No

### Microsatellite analysis

Genetic diversity at eight microsatellite loci was analyzed to differentiate selective and demographic influences on the MHC class IIB alleles in the studied stickleback populations. The mean observed heterozygosities (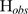) were variable among populations, and showed a slight excess of homozygosity relative to Hardy Weinberg expectations (Table 1). Limnetic and benthic populations had similar levels of allelic richness (Rs) in the same lake (Table 1), and neither locus-specific Rs values (two-tailed permutation test, 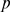 = 0.74) nor population-specific Rs values (Friedman-test, 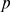 = 0.20) were significantly different between limnetics and benthics within a lake. At the individual level, the mean number of microsattelite alleles per locus (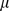
Sats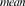) was not different between limnetics and benthics in both Priest (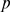 = 0.16) and Paxton Lake (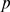 = 0.28) (Table 1). Similarly, expected heterozygosity (H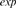) was similar among the studied populations, and was not significantly different between limnetics and benthics (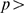
0.05). Hence, our results suggest that the greater number of MHC alleles in benthics compared to limnetics is not a result of differences in effective population size or neutral processes such as drift.

### MHC simulation model results

**Figure 3:**
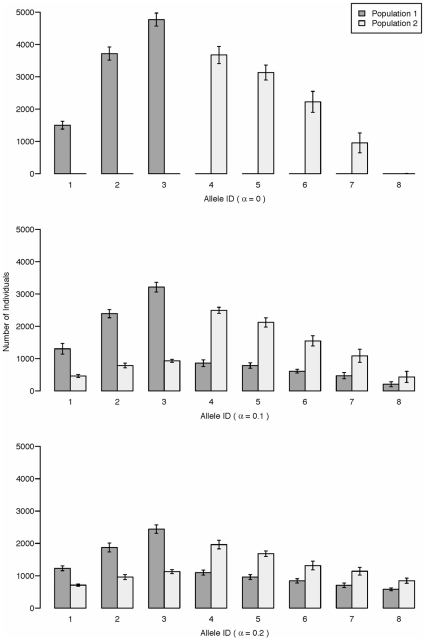
Simulation output with varying levels of assortative mating. Bar heights are the mean allele counts averaged over 10 simulations, and error bars denote one standard deviation. Simulations were run for 1000 generations with 10,000 individuals in each population. All alleles were initially present at equal frequencies. Selection strengths were 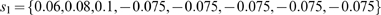 and 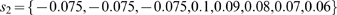 and recombination occurs freely between all loci.

**Figure 4:**
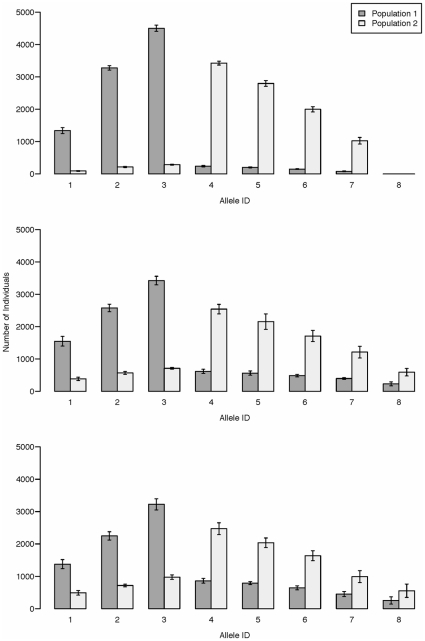
Simulation output with varying selection strengths. Bar heights are the mean allele counts averaged over 10 simulations, and error bars denote one standard deviation. Simulations were run for 1000 generations with 10,000 individuals in each population. All alleles were initially present at equal frequencies. 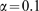 in all plots and recombination occurred freely between all loci. Selection strengths were, in the top plot: 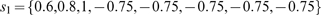 and 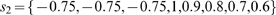, in the middle plot: 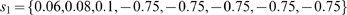 and 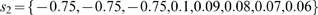, and in the bottom plot: 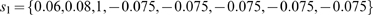 and 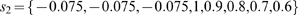.

The divergence in MHC allele frequencies that we observed between limnetics and benthics (Figure 2) is consistent with the patterns seen from our simulations when there is introgression between species foraging in contrasting selective environments (Figure 3, 4). When specific MHC alleles confer fitness advantages in either the benthic or pelagic foraging habitat, we observed divergence in the composition of MHC alleles between limnetic and benthic stickleback that is driven by frequency dependent selection (Figure 3). The frequency of alleles that are shared by both the benthic and limnetic species increases with higher levels of hybridization (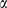 increases from 0–0.2; Figure 3). Hence, alleles could persist in the limnetic species, for example, that only confer benthics with resistance to benthic parasites purely as a consequence of introgression between the species. In contrast, increasing the intensity of selection against maladapted alleles in one habitat will reduce the amount of allele sharing between species (Figure 4). Overall, our simulations show that the rate of hybridization between species and the strength of selection between habitats will jointly determine the amount of overlap in the frequency distributions of MHC alleles between benthic and limnetic stickleback.

## Discussion

We found divergence in MHC genes between closely related stickleback species and populations that forage in contrasting habitats (Figure 1). The parallel divergence in the number of MHC class II alleles per individual between limnetic and benthic species in two lakes with species pairs is not observed in the allelic richness of microsatellites, and is unlikely to have occurred by drift. We also observed a similar pattern of divergence in the individual MHC allelic richness between two stickleback populations with a littoral and pelagic phenotype. Overall, these results suggest a correlation between MHC genotype and foraging habitat, whereby stickleback have lower individual MHC allelic richness in pelagic compared to littoral foraging habitats. MHC divergence between limnetics and benthics is a key prediction from a recent model of MHC-based pleiotropic speciation in stickleback [12], and likely results from contrasting parasite communities in pelagic and littoral habitats [25].

### What explains variation in allelic richness of MHC genes in stickleback?

Local scale heterogeneity in the distribution of parasite communities [24], [25] could help explain the high levels of MHC diversity in natural populations [8]. The spatial scale of parasite heterogeneity, along with the frequency of host-parasite interactions, will determine whether parasites cause balancing or divergent selection on their hosts [17]. Our results suggest that genetic diversity at MHC loci in freshwater fish could be promoted by the contrasting parasite communities in benthic and pelagic habitats of lakes [25]. In natural systems, an individual's immune system is persistently challenged by multiple parasites that vary widely in their virulence [17]. Host-parasite co-evolution occurs independently between each species of parasite and host, meaning that parasite communities might maintain a constant diversity of challenges to a host's immune system over time without causing dramatic temporal fluctuations in gene frequencies [17]. If contrasting co-evolutionary dynamics are occuring in adjacent habitats, then rare MHC alleles might be maintained in the population if they are selectively neutral in some environments, but beneficial in others. Indeed, our simulations predict that the extent of allele sharing between sympatric host populations should depend strongly on the contrasting selection pressures that are mediated by parasites in adjacent foraging environments (Figure 3). An important next step, is to experimentally confirm that contrasting parasite communities in lakes can cause disruptive selection on stickleback populations, as has been shown in other host-parasite systems [9].

Our results suggest that the optimal number of MHC alleles per individual depends on habitat specialization by stickleback (Figure 1). This conclusion is supported by a similar pattern of divergence in the individual MHC allelic richness between lake and river stickleback populations in Northern Germany [15]. River stickleback, compared to lake stickleback, have a lower number of MHC alleles per individual fish [15], have a lower parasite load [22], and are less resistant to lake parasites [34]. The associations between host genotype and parasite resistance are currently unknown in the benthic-limnetic system, and so the underlying mechanism of MHC divergence is still uncertain. Nevertheless, our results for stickleback, along with those for cichlids [28], provide good evidence for MHC divergence between closely related species that live in the same lake but exploit different foraging habitats [28].

### How might parasites cause divergence in the number of MHC alleles?

**Figure 5:**
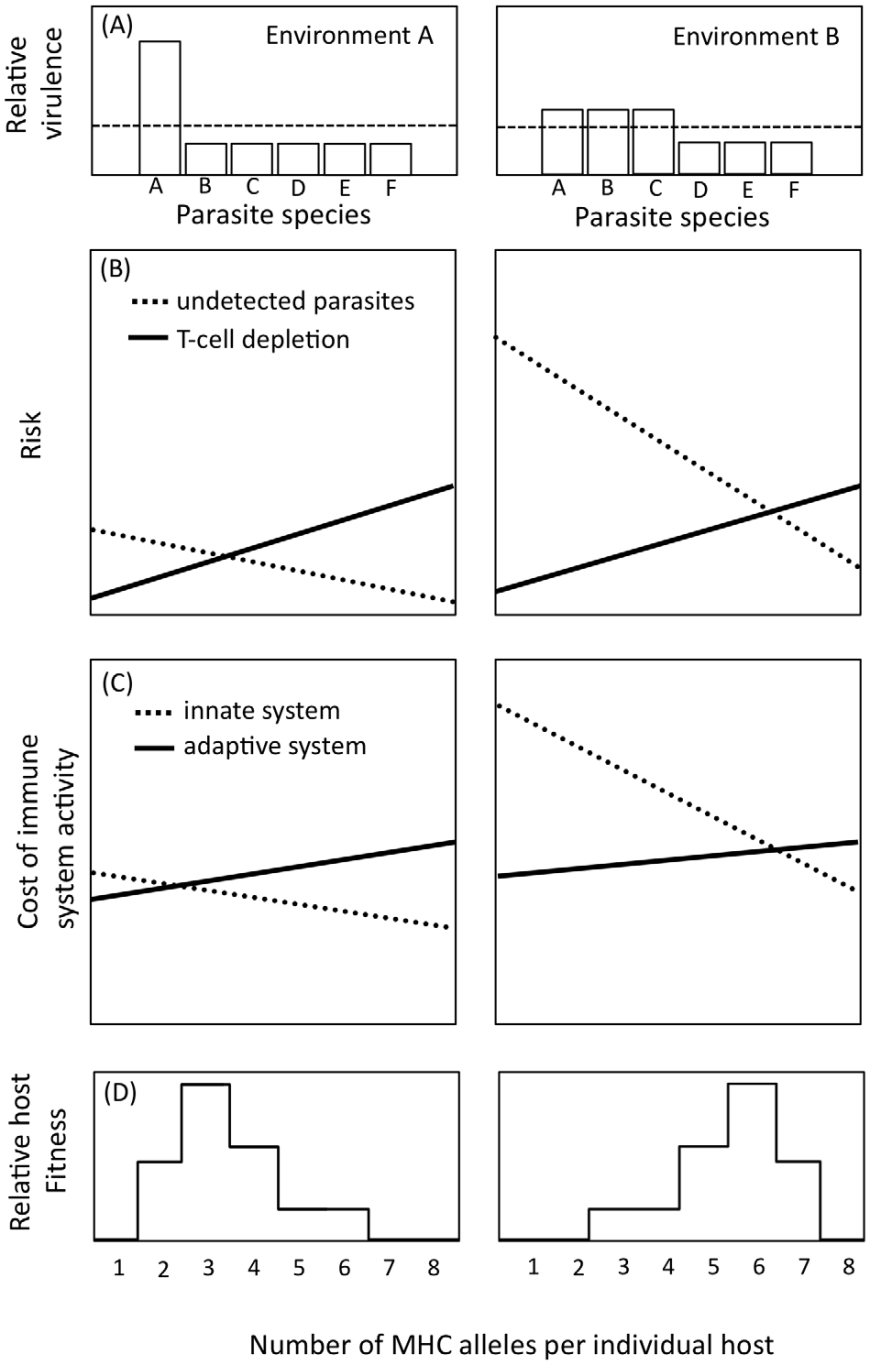
Two scenarios leading to divergent selection on MHC genotype. Panel (A) shows the contrasting virulence distribution of two parasite communities, where the dotted line indicates that the average virulence of the community is similar in both environments. Panel (B) shows the tradeoff between the ability of the immune system to detect parasites (dotted lines) and the risk of T-cell depletion (solid lines). Panel (C) shows the tradeoff between allocating resources to the adaptive versus the innate immune system. Panel (D) illustrates the resulting population distribution of individual allele number in the two environments (A or B) resulting from divergent selection mediated by either of the tradeoffs illustrated in Panel B and C.

Parasites can drive host evolution in several ways [35], but here we consider two possibilities for how contrasting parasite communities (Figure 5, Panel A) might generate divergent selection on hosts culminating in evolution in the number of alleles per individual (Figure 5, Panel D).

The first possibility is that hosts may experience different levels of risk associated with detecting parasite infections in environments with contrasting parasite communities (Figure 5, Panel B, dotted line shows the declining risk of not detecting parasites). In this case, the adaptive immune system's ability to detect parasites tends to increase with an increasing number of MHC alleles [36] (Figure 5B). Prevailing theory suggests that an intermediate number of MHC alleles is optimal, because too few alleles reduces the detection rate of antigens by T-cells, and too many alleles increases the likelihood of T-cell depletion [36], [37]. T-cells undergo a process called negative selection during their maturation in the thymus, which reduces the number of self-reactive MHC molecules and the likelihood of autoimmune diseases [36]. However, negative selection can compromise immune responses when the number or diversity of T-cells is severely reduced (i.e. T-cell depletion). Natural selection on the stringency of this negative selection process is thought to explain most of the natural variation in the allelic repertoire of individuals among systems [22], [37], but it is largely unknown how the risk of T-cell depletion might differ between environments [37]. In fact, [38] suggests that the risk of T-cell depletion, illustrated as a linear function in Figure 5 (solid line), may only be expressed at very high levels of allelic diversity that is not observed in nature. If so, T-cell depletion would not be a likely cause for the low levels of individual allelic richness in limnetics, and would probably not cause the observed divergence in allele number between sympatric species. Nevertheless, assuming the risks of T-cell depletion are similar between environments and positively related with MHC allelic richness (Figure 5B), divergent selection could arise if hosts experience different levels of parasite risk in adjacent foraging habitats (Figure 5B, compare two dotted lines). A limitation of this theory, however, is that is does not consider the relative costs of the innate and adaptive components of the immune system in different ecological contexts, so it might inadequately explain allelic variation of MHC over a broad range of natural environments [16], [17].

A second possibility is that hosts adopt different allocation strategies to immune defense in environments with contrasting parasite communities [16]–[18]. In this case (Figure 5C), the evolution of host specificity in immune defense (i.e. adaptive immune system) occurs at the expense of allocation to a generalist strategy (i.e. innate immune system) [18]. Investing resources into the adaptive immune system is costly (Figure 5C, solid line), but reduces the maintenance costs of the innate system (Figure 5C, dotted line). Because the innate system compensates for the adaptive system's deficiencies in coping with multiple virulent parasites, the benefits of an efficient innate system are greatest in risky environments (e.g. Figure 5, Environment B). In such environments, individuals with few alleles will have a low fitness, either because they receive more parasite infections or because they must up-regulate their innate immune system. In comparison, in environments with few virulent parasites (e.g. Figure 5, Environment A), hosts are better off having fewer MHC alleles, and possibly multiple copies of them [39], for targeting the few virulent parasites in the environment.

We believe that our understanding about the role of parasitism in host speciation will be improved by studying the mechanisms of how parasites might cause divergent selection on host traits. In stickleback, and other freshwater fish, more research is needed to understand how the distribution of parasite communities in pelagic and benthic environments can affect both the innate and adaptive immune systems of hosts.

### Implications of MHC divergence for the magic-trait model of ecological speciation

Functional traits under disruptive selection that also form the basis of assortative mating have been dubbed ‘magic’ because of their potential to accelerate speciation in sympatry [11], [40]. In threespine stickleback three ‘magic traits’ have been proposed, namely body size at maturity [14], feeding behavior [41], and MHC genotype [12].

Body size is a good candidate for a magic trait in stickleback [14], because it is commonly under divergent selection in nature [1], [42], and extensive mating trials, particularly between benthics and limnetics [43], show that body size strongly underlies stickleback mate choice [44]. However, the source of divergent natural selection on stickleback body size could result from tradeoffs related to both feeding performance [41], [45] and parasite infection [14], [46]. Stickleback body size and feeding behavior, for example, are often correlated in stickleback populations [47], and so assortative mating may appear to reflect recent feeding history [41] even if body size is the proximate cue for mate choice [43]. Correlations between body size and MHC genotype (two putative magic-traits) may also exist within stickleback populations [46], which is intriguing because peptides originating from MHC genes could also be associated with odors [27] that influence mating decisions [31]. To date, MHC-based mate choice has not been investigated for benthic and limnetic sticklebacks, and so more experimental tests would be useful to disentangle the effects of body size and MHC genotype on stickleback mating preferences.

In summary, our results demonstrate that limnetics and benthics have divergent MHC genotypes in a pattern consistent with the divergence also observed between populations along the pelagic-littoral gradient (Figures 1). It is still an open question whether these MHC differences between species are a by-product of habitat specialization, and whether they underly pleiotropic speciation in stickleback [12]. Future studies should experimentally test for parasite-mediated disruptive selection on MHC genotypes (Figure 5), and examine MHC based mate choice between species and populations with contrasting parasite exposure.

## Materials and Methods

### Study sites and collections

We sampled stickleback from four lakes: Cranby Lake (CRA), Paxton Lake (PAX), Priest Lake (PRI), and Kennedy Lake (KEN). Located on Texada Island, Paxton (Depth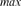 = 13, Area = 17 ha) and Priest Lake (Depth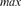 = 17.3, Area = 44.3 ha) have sympatric species of limnetic and benthic sticklebacks. The nearby Cranby Lake has a similar morphometry (Depth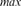 = 12.3, Area = 44.6 ha) to Paxton and Priest Lakes, but has an allopatric stickleback population that is intermediate in morphology and diet between limnetics and benthics [48]. In contrast to these systems, Kennedy Lake is a large deep lake on the West Coast of Vancouver Island (Depth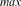 = 145, Area = 6475 ha), and has a population of sticklebacks with a pelagic phenotype. Overall, our study lakes include two lakes with a benthic and limnetic species pair (Paxton and Priest), one population with an intermediate phenotype (Cranby), and one population with a pelagic phenotype from a large and deep lake (Kennedy). We set minnow traps overnight to collect sticklebacks from the littoral habitats during the breeding season (May–July). Limnetic and benthic species were differentiated based on phenotypic differences in body amour and shape. All fish were immediately frozen after collection, and subsequently stored at −80°C until analysis.

### MHC analysis

The exact genomic structure of the stickleback MHC is still unknown, but partial regions of MHC class I and class II have been described previously [49], [50]. In this study, we focused on exon 2 which encodes the peptide-binding region (B1 domain) and presents the most polymorphic part of the class IIB genes. We used capillary electrophoresis single-strand conformation polymorphism (CE-SSCP) to screen and identify the allelic variants of the MHC class IIB genes in a larger number of samples. This method allows for high throughput, high sensitivity and good reproducibility [51], [52]. We use the term ‘allelic richness’, as opposed to ‘allelic diversity’ [53], to describe the number of MHC class II alleles in either a stickleback population or in an individual.

Genomic DNA was extracted from tissue samples (fin clips) with a DNA extraction kit DNeasy Tissue Kit (Qiagen GmbH, Hilden, Germany) according to the manufacturers protocol. The Qiagen Multiplex PCR Kit (Qiagen GmbH, Hilden, Germany) with fluorescent labeled primers (forward primer by 6-FAM and reverse primer by NED) was used to amplify exon 2. We used GA11 as our forward primer [54], which hybridizes in most bony fish in the much conserved 5 area of the exon 2. We also used a new reverse primer (GA11R 5 GAC TCA CCG GAC TTA GTC AG 3) that we designed based on available published stickleback MHC class IIB sequences [50], [55] and MHC class IIB sequences that were obtained from the stickleback Ensemble Genome Browser.

The thermal cycling profile for the PCRs consisted of initial heating at 95°C for 15 min (hot-start polymerase activation), followed by 30 cycles of denaturation at 94°C for 30 sec, annealing at 56°C for 30 sec, extension at 72°C for 90 sec, and ending with a 10 min extension step at 72°C. The isolated fragment length was 242bp (including primer sequences) and parts of it have been previously characterized [50]. For the CE-SSCP analyses, the fluorescent-labeled PCR samples were prepared for electrophoresis by combining 1 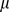
L PCR product with 14 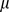
L loading mix which consisted of 13.75 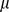
L Hi-DI formamide and 0.25 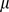
L Genescan ROX 350 standard (Applied Biosystem). The mixture was heated for 3 min at 95°C to separate the complementary DNA strands, chilled on ice for 4 min and analyzed by capillary electrophoresis on an ABI PRISM ®3100 automated DNA Sequencer (Applied Biosystem). The CE-SSCP polymer consisted of 5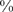 Genescan polymer (Applied Biosystem), 10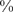 glycerol, 1×TBE, and HPLC-water. The running buffer mixture contained 10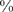 glycerol, 1×TBE and HPLC-water. The separation of the allelic variants was achieved by run conditions at 12kV for 36 minutes and by a run temperature at 22°C. The retention times of the sequence variants were identified relative to the ROX 350 standard. The GeneMapper software packages 4.05 from Applied Biosystems were used to process the obtained SSCP data. With this PCR and screening approach we detected between one and eight sequence variants per individual which probably reflects the previously estimated number of genes (i.e. 3 to 4) per individual [55], [56].

### Microsatellite analysis

We used eight microsatellite loci to compare genetic variation among stickleback populations: Gac1125, Gac5017, Gac4174, Gac2111, Gac7188, Gac1097, Gac7033, and Gac5196 [57], [58]. All PCRs were carried out in 12 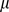
L reaction volumes. Each PCR reaction contained one to two microlitre genomic DNA, fluorescent labelled (Applied Biosystems) primers and the Qiagen Multiplex PCR Kit (Qiagen GmbH, Hilden, Germany). The PCR programme used was 15 min 95°C followed by 35 cycles of 30 s at 94°C, 90 s annealing and 90 s at 72°C, ending with a 10 min final elongation stage at 72°C. The annealing temperatures for the multiplex PCRs (two microsatellite loci were run in the same multiplex PCR) were 59°C for the microsatellites Gac1125 and Gac5017; 57°C for Gac4174 and Gac2111; Gac7188 and Gac1097; Gac7033 and Gac5196. PCR products were separated and scored on an ABI PRISM 3100 automated DNA Sequencer (Applied Biosystem). Using these markers we calculated the mean number of alleles, observed heterozygosity (H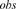) and expected heterozygosity (H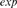) using the Arlequin [59] and Genetix 4.02 [60] software. We calculated the mean number of microsattelite alleles per locus (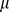
Sats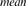) to compare with the allelic richness at MHC loci (i.e. MHC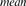), and tested for differences between benthics and limnetics. In addition, we used FSTAT, version 2.9.3 [61] to calculate locus-specific values of allelic richness (Rs), which is based on a rarefaction approach and accounts for unbalanced sample sizes. We calculated population-specific Rs values as arithmetic means over all polymorphic loci, and used the Friedman-test, a non-parametric test, to compare Rs values among stickleback populations. Using FSTAT we tested for significant (1000 permutations) differences in Rs between the limnetic and benthic populations.

### Statistical analysis

We used log-linear analysis to examine how the number and frequency of MHC alleles differed among lake populations and ecotypes (Table 1). To simplify the interpretation of these analyses, we compared allele frequencies of stickleback from both sympatric lakes (Paxton and Priest) to stickleback from a single allopatric lake (Cranby Lake). We formulated seventeen different linear models (Table 2), based on seven different questions (Table 3), and then used AIC to select the model best supported by our data. We used this approach to determine how the average number of alleles and the frequency of specific alleles differed among lake populations and stickleback ecotypes (Table 2). All statistical analyses were done using R [62].

### MHC simulation model

We constructed a simulation model to examine whether introgression between limnetics and benthics, along with contrasting selective environments in adjacent foraging habitats, could explain the observed distribution of MHC alleles in Paxton and Priest Lake. Because of several uncertainties, including not knowing the exact number of MHC loci in stickleback and not being able to quantify copy number variation using CE-SSCP, we did not use the model to estimate the strength of selection at MHC loci. Instead, we used it to illustrate how variation in the strength of selection and the level of assortative mating could affect the frequency distribution of MHC alleles in benthics and limnetics.

We modeled stickleback recombination using a haploid model, partly because our MHC typing method cannot distinguish between homozygotes and heterozygotes at a particular MHC locus. We considered two haploid populations (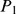 and 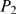) of size 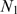 and 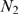. Each life cycle consists of selection followed by reproduction, and population sizes are held constant throughout. Population identity is determined by a single locus with two alleles, and we assumed that the two populations preferentially occupy different environments; that is, the species identity locus codes for habitat preference. A second locus with 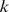 alleles controls fitness with respect to MHC haplotype in each environment. The selection coefficients of alleles in each environment are given by the 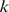-dimensional vectors 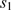 and 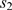, and selection in each environment is assumed to be frequency-dependent.

To illustrate with an example, suppose we have 3 alleles at the MHC locus and 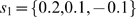. This implies that alleles 1 and 2 are favoured in environment 1. To compute the relative fitnesses of each of these alleles in environment 1 selection coefficients were weighted by allele frequencies, as rare alleles are assumed to experience a frequency dependent advantage. Letting 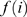 denote the frequency of allele 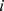, the fitnesses of the three alleles in environment 1 are then 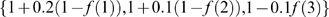, in the order given. Because we are assuming that common MHC alleles are more easily recognized by parasites, the above implementation results in beneficial alleles experiencing the strongest positive selection pressure when they are rare (e.g., 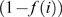 is large), with the strength of selection decreasing as they become more abundant (e.g., as 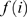 increases). Similarly, deleterious alleles experience the strongest selection pressure against them when they are common. Recombination occurs between the two loci at rate 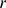. Individuals mate within their population with probability 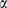 and “hybridize” with individuals from the other population with probability 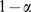. Thus 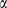 can be viewed as the probability of mating assortatively with respect to species identity.

## References

[1] Schluter D (2000). The ecology of adaptive radiation.

[2] Rundle HD, Nosil P (2005). Ecological speciation.. Ecology Letters.

[3] Buckling A, Rainey PB (2002). The role of parasites in sympatric and allopatric host diversification.. Nature.

[4] Summers K, McKeon S, Sellars J, Keusenkothen M, Morris J (2003). Parasitic exploitation as an engine of diversity.. Biological Reviews.

[5] Nuismer SL, Otto SP, Blanquart F (2008). When do host-parasite interactions drive the evolution of non-random mating?. Ecology Letters.

[6] Poulin R, Thomas F (1999). Phenotypic variability induced by parasites: Extent and evolutionary implications.. Parasitology Today.

[7] Koskella B, Lively CM (2009). Evidence for negative frequency-dependent selection during experimental coevolution of a freshwater snail and a sterilizing trematode.. Evolution.

[8] Wegner KM (2008). Historical and contemporary selection of teleost MHC genes: did we leave the past behind?. Journal of Fish Biology.

[9] Duffy MA, Brassil CE, Hall SR, Tessier AJ, Caceres CE (2008). Parasite-mediated disruptive selection in a natural *Daphnia* population.. BMC Evolutionary Biology.

[10] Violle C, Navas ML, Vile D, Kazakou E, Fortunel C (2007). Let the concept of trait be functional!. Oikos.

[11] Gavrilets S (2004). Fitness landscapes and the origin of species.

[12] Eizaguirre C, Lenz TL, Traulsen A, Milinski M (2009). Speciation accelerated and stabilized by pleiotropic major histocompatibility complex immunogenes.. Ecology Letters.

[13] Hamilton WD, Zuk M (1982). Heritable true fitness and bright birds - a role for parasites.. Science.

[14] MacColl ADC (2009). Parasites may contribute to ‘magic trait’ evolution in the adaptive radiation of three-spined sticklebacks, Gasterosteus aculeatus (*Gasterosteiformes: Gasterosteidae*).. Biological Journal of the Linnean Society.

[15] Milinski M (2006). The major histocompatibility complex, sexual selection, and mate choice.. Annual Review of Ecology and Systematics.

[16] Frank SA (2000). Specific and non-specific defense against parasitic attack.. Journal of Theoretical Biology.

[17] Jokela J, Schmid-Hempel P, Rigby MC (2000). Dr. Pangloss restrained by the Red-Queen - steps towards a unified defence theory.. Oikos.

[18] Sadd BM, Schmid-Hempel P (2009). Principles of ecological immunology.. Evolutionary Applications.

[19] Medzhitov R, Janeway CA (2002). Decoding the patterns of self and nonself by the innate immune system.. Science.

[20] Huges AL, Nei M (1989). Nucleotide substitution at major histocompatability complex Class-II loci - Evidence for overdominant selection.. Proceedings of the National Academy of Sciences.

[21] Moret Y, Schmid-Hempel P (2000). Survival for immunity: The price of immune system activation for bumblebee workers.. Science.

[22] Scharsack JP, Kalbe M, Harrod C, Rauch G (2007). Habitat-specific adaptation of immune responses of stickleback *Gasterosteus aculeatus* lake and river ecotypes.. Proceedings of the Royal Society B-Biological Sciences.

[23] Reimchen T, Nosil P (2001). Ecological causes of sex-biased parasitism in threespine stickleback.. Biological Journal of the Linnean Society.

[24] Knudsen R, Prmicerio R, Amundsen P, Klemetsen A (2009). Temporal stability of individuals feeding specialization may promote speciation.. Journal of Animal Ecology.

[25] MacColl ADC (2009). Parasite burdens differ between sympatric three-spined stickleback species.. Ecography.

[26] Thompson JN (2009). The coevolving web of life.. American Naturalist.

[27] Milinski M, Griffiths S, Wegner KM, Reusch TBH, Haas-Assenbaum A (2005). Mate choice decisions of stickleback females predictably modified by MHC peptide ligands.. Proceedings of the National Academy of Sciences.

[28] Blais J, Rico C, van Ossterhout C, Cable J, Turner GF (2007). MHC Adaptive divergence between closely related and sympatric African Cichlids.. PLoS ONE.

[29] Reimchen T, Nosil P (2001). Lateral plate asymmetry, diet and parasitism in threespine stickleback.. Journal of Evolutionary Biology.

[30] Wegner KM, Reusch TBH, Kalbe M (2003). Multiple parasites are driving major histocompatibility complex polymorphism in the wild.. Journal of Evolutionary Biology.

[31] Reusch T, Haberli M, Aeschlimann P, Milinski M (2001). Female sticklebacks count alleles in a strategy of sexual selection explaining MHC polymorphism.. Nature.

[32] Lavin PA, McPhail JD (1986). Adaptive divergence of trophic phenotype among fresh-water populations of the threespine stickleback *(Gasterosteus-aculeatus)*.. Canadian Journal of Fisheries and Aquatic Sciences.

[33] Schluter D (1993). Adaptive radiation in sticklebacks - size, shape, and habitat use efficiency.. Ecology.

[34] Kalbe M, Kurtz J (2006). Local differences in immunocompetence reflect resistance of sticklebacks against the eye fluke *Diplostomum pseudospathaceum*.. Parasitology.

[35] Schulenburg H, Kurtz J, Moret Y, Siva-Jothy MT (2009). Introduction. Ecological immunology.. Philosophical transactions of the Royal Society B- Biological Sciences.

[36] Nowak MA, Tarczyhornoch K, Austyn JM (1992). The optimal number of major histocompatability complex molecules in an individual.. Proceedings of the National Academy of Sciences.

[37] Woelfing B, Traulsen A, Milinski M, Boehm T (2009). Does intra-individual major histocompatibility complex diversity keep a golden mean?. Philosophical Transactions of the Royal Society B- Biological Sciences.

[38] Borghans JAM, Noest AJ, Boer RJD (2003). Thymic selection does not limit the individual MHC diversity.. European Journal of Immunology.

[39] Traherne JA (2008). Human MHC architecture and evolution: implications for disease association studies.. Internatonal Journal of immunogenetics.

[40] Dieckmann U, Doebeli M (1999). On the origin of species by sympatric speciation.. Nature.

[41] Snowberg LK, Bolnick DI (2008). Assortative mating by diet in a phenotypically unimodal but ecologically variable population of stickleback.. American Naturalist.

[42] Bolnick DI, Lau OL (2008). Predictable patterns of disruptive selection in stickleback in postglacial lakes.. American Naturalist.

[43] Nagel L, Schluter D (1998). Body size, natural selection, and speciation in sticklebacks.. Evolution.

[44] McKinnon JS, Mori S, Blackman BK, David L, Kingsley DM (2004). Evidence for ecology's role in speciation.. Nature.

[45] Schluter D (1995). Adaptive radiation in sticklebacks - trade-offs in feeding performance and growth.. Ecology.

[46] Eizaguirre C, Yeates SE, Lenz TL, Kalbe M, Milinski M (2009). MHC-based mate choice combines good genes and maintenance of MHC polymorphism.. Molecular Ecology.

[47] Matthews B, Marchinko KB, Bolnick DI, Mazumder A (2010). Specialization of trophic position and habitat use by sticklebacks in an adaptive radiation.. Ecology.

[48] Robinson BW (2000). Trade offs in habitat-specific foraging efficiency and the nascent adaptive divergence of sticklebacks in lakes.. Behaviour.

[49] Schaschl H, Wegner KM (2007). Contrasting mode of evolution between the MHC Class I genomic region and Class II region in the three-spined stickleback (*Gasterosteus aculeatus L.; Gasterosteidae : Teleostei*).. Immunogenetics.

[50] Reusch TBH, Schaschl H, Wegner KM (2004). Recent duplication and inter-locus gene conversion in major histocompatibility Class II genes in a Teleost, the three-spined stickleback.. Immunogenetics.

[51] Binz T, Reusch TBH, Wedekind C, Milinski M (2001). SSCP analysis of MHC Class IIB genes in the threespine stickleback.. Journal of Fish Biology.

[52] Schaschl H, Wegner KM (2006). Polymorphism and signature of selection in the MHC class I genes of the three-spined stickleback *Gasterosteus aculeatus*.. Journal of Fish Biology.

[53] Wegner KM, Kalbe M, Kurtz J, Reusch TBH, Milinski M (2003). Parasite selection for immunogenetic optimality.. Science.

[54] Sato A, Figueroa F, Murray BW, Malaga-Trillo E, Zaleska-Rutczynska Z (2000). Nonlinkage of major histocompatibility complex class I and class II loci in bony fishes.. Immunogenetics.

[55] Reusch TBH, Langefors A (2005). Inter- and intralocus recombination drive MHC class IIB gene diversification in a teleost, the three-spined stickleback *Gasterosteus aculeatus*.. Journal of Molecular Evolution.

[56] Hohenlohe PA, Bassham S, Etter PD, Stiffler N, Johnson EA (2010). Population genomics of parallel adaptation in threespine stickleback using sequenced RAD tags.. PLoS Genetics.

[57] Heckel G, Zbinden M, Mazzi D, Kohler A, Reckeweg G (2002). Microsatellite markers for the three-spined stickleback *(Gasterosteus aculeatus L.)* and their applicability in a freshwater and an anadromous population.. Conservation Genetics.

[58] Largiader CR, Fries V, Kobler B, Bakker TCM (1999). Isolation and characterization of microsatellite loci from the three-spined stickleback *(Gasterosteus aculeatus L.)*.. Molecular Ecology.

[59] Excoffier L, Laval G, Schneider S (2005). Arlequin: An integrated software package for population genetics data analysis.. Evolutionary Bioinformatics.

[60] Belkhir K, Castric V, Bonhomme F (2002). Identix, a software to test for relatedness in a population using permutation methods.. Molecular Ecology Notes.

[61] Goudet J (1995). FSTAT (Version 1.2): A computer program to calculate F-statistics.. Journal of Heredity.

[62] RDevelopmentTeam (2008). R: A Language and Environment for Statistical Computing.. http://www.R-project.org.

